# Residuals of Chemical Cleaning Agents Impair Peri-Implant Cell Viability: An in Vitro Study

**DOI:** 10.1021/acsbiomaterials.5c01777

**Published:** 2026-01-13

**Authors:** Qiang Wang, Håvard Jostein Haugen, Dirk Linke, Ståle Petter Lyngstadaas, Qianli Ma

**Affiliations:** † Department of Biomaterials, Faculty of Dentistry, 227896 University of Oslo, Oslo 0455, Norway; ‡ Department of Biosciences, Faculty of Natural Sciences, University of Oslo, Oslo 0316, Norway

**Keywords:** chemical debridement agents, residuals, cytocompatibility, regeneration, apoptosis

## Abstract

Background: Chemical debridement agents are commonly used during the cleaning of implants for peri-implantitis treatment; however, how these agents affect lesion healing remains unclear. In addition, the dose- and time-dependent effects of these residuals on implant biocompatibility remain poorly understood. Materials and methods: We evaluated the effects of active compounds in commercial products-3% hydrogen peroxide (H_2_O_2_), 0.43% sodium hypochlorite (NaClO), and 0.12% chlorhexidine with 0.05% cetylpyridinium chloride (CHX-CPC) at graded dilutions on murine osteoblastic cells (MC3T3-E1), human gingival fibroblasts (HGFs), and human bone marrow mesenchymal stromal cells (hBMSCs). Cells were cultured for 24 h, then exposed to the agents for 2, 12, or 24 h. Cytotoxicity and viability were assessed using lactate dehydrogenase (LDH) release and CCK-8 assays, while cell morphology was examined by scanning electron microscopy (SEM). Apoptotic gene expression (*BCL2*, *MCL1*, *BAX*) was analyzed after 2 h using quantitative PCR. Results: At high concentrations, H_2_O_2_ and NaClO significantly reduced LDH activity in supernatant, likely due to oxidant-induced enzyme inactivation. All three agents inhibited cell viability in a dose- and time-dependent manner, accompanied by cell shrinkage and deformation. Among the tested cell types, hBMSCs displayed greater resistance to H_2_O_2_, maintaining proliferative viability at 0.15% (1:20 dilution). Gene expression analysis revealed that concentrated H_2_O_2_ and CHX-CPC downregulated *BCL2* and *MCL1* expression in MC3T3-E1 cells, with broader suppression of these genes observed in HGFs across all agents. In hBMSCs, high concentrations of the agents did not significantly reduce *BCL2* and *MCL1* levels. Conclusion: Residual chemical debridement agents, when inadequately removed, compromise the viability of cells in peri-implant tissues in a dose- and time-dependent manner. hBMSCs exhibited greater resistance to apoptosis than MC3T3-E1 cells and HGFs. Thorough removal of residual chemical cleaning agents after peri-implant debridement is therefore crucial to preserve the biocompatibility of the implant and the healing potential of peri-implant tissues.

## Graphical abstract

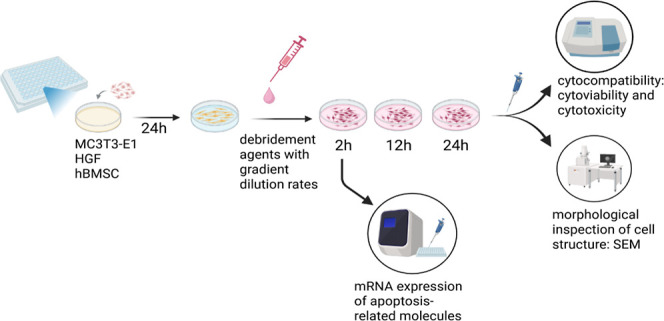

## 1 Introduction

The long-term success of dental implants depends not only on stable primary osseointegration but also on robust, stable, and long-lasting soft-tissue integration at the transgingival region. This soft-tissue integration is hindered by the continuing growth of oral biofilms on the implant surface, which can trigger chronic inflammatory responses.^1^ Accordingly, sustained peri-implant health requires both periodical disruption of the microbial biofilm to prevent pathogenic colonization and reliable achievement of osseointegration and soft-tissue integration after surgery.^2^ Numerous nonsurgical debridement strategies have been proposed to eliminate biofilms from implant surfaces, yet no consensus has been reached regarding the superiority of any single method.^3,4^ Among these approaches, chemical agents such as chlorhexidine (CHX), citric acid, sodium hypochlorite (NaClO), and H_2_O_2_ are widely employed for implant surface decontamination. While these agents are effective in killing bacteria and/or removing biofilm, their biological effects on cells in peri-implant tissues remain incompletely understood.^5,6^

In our previous work, we investigated the cytocompatibility of commonly used chemical debridement agents on titanium implant surfaces, including H_2_O_2_, Poloxamer, Perisolv (0.43% NaClO), and Paroex (0.12% CHX +0.05% cetylpyridinium chloride, CPC). Peri-implant tissues comprise diverse cell populations, including immune, mesenchymal, epithelial, and fibroblast lineages.^7^ To represent the key cellular compartments essential for peri-implant regeneration, we selected human bone marrow mesenchymal stem cells (hBMSCs), as progenitors of osteoblasts that are critical for osseointegration,^8^ and human gingival fibroblasts (HGFs), the main cellular component of peri-implant connective tissue that forms the protective soft-tissue seal at the transmucosal region.^2^ In addition, we included the murine preosteoblastic MC3T3-E1 cells, which exhibit proliferative and mineralization behaviors comparable to those of human primary osteoblasts.^9^ Our findings revealed that the adhesion and proliferation of hBMSCs were significantly impaired on commercial titanium implant surfaces (OsseoSpeed) after treatment with 3% H_2_O_2_ and commercial formulations such as Perisolv (0.43% NaClO) and Paroex (0.12% CHX +0.05% CPC). Moreover, oxidant-based agents (H_2_O_2_ and NaClO) suppressed key genes associated with proliferation, antiapoptosis, and cellular attachment to titanium.^10^ These results highlight the potential adverse cellular effects of chemical cleaning procedures and emphasize the importance of adequate post-treatment rinsing to minimize residual toxicity.

Although residues of chemical debridement agents can persist around implants after treatment,^11^ the dose- and time-dependent effects of these agents on peri-implant tissue regeneration remain incompletely elucidated. To address this knowledge gap, we systematically evaluated and analyzed the biological impact of residual active components from widely used debridement agents-H_2_O_2_, NaClO, and CHX-CPC on MC3T3-E1, HGFs, and hBMSCs to inform safer protocols that minimize cytotoxicity and preserve a regenerative peri-implant environment, thereby supporting the long-term success of implant treatment. To simulate these clinical conditions in which traces of these agents may remain in peri-implant pockets, test solutions were prepared at graded dilutions (1:2000, 1:500, 1:200, 1:100, 1:50, 1:20, and 1:5) and applied to cells for 2, 12, or 24 h. Cytocompatibility (cytotoxicity and proliferative viability) and apoptosis-related gene expressions (*BCL2*, *MCL1*, *BAX*) were subsequently examined to characterize cellular responses.

## 2 Methods and Materials

### 2.1 Chemical Debridement Agent

The debridement chemicals used included hydrogen peroxide (H_2_O_2_; Cat. No 1.08597, Sigma-Aldrich, Germany), sodium hypochlorite (NaClO; Cat. No 87939.29, VWR, Germany), chlorhexidine digluconate (CHX; aqueous solution, Cat. No 41385.AC, VWR, Germany) and cetylpyridinium chlorite (CPC, Cat. No ACRO226991000, VWR, Belgium). The concentrations of the different debridement chemicals were selected based on those found in the commercial products Perisolv (Regedent AG) and Gum Pareox (Sunstar). Stock solutions were diluted in culture medium supplemented with 10% FBS (fetal bovine serum, Cat. No. A5256701, Gibco, NY, USA) or platelet lysate (PIPL, kindly donated by the Blood Bank, Landspitali University Hospital, Reykjavik, Iceland) to obtain graded concentrations as shown in Table 1.

**Table 1:** Chemical Debridement Agents Used and Dilution Ratios

commercial debridement agent	active chemical compounds	dilution ratios and concentrations						
		1:2000	1:500	1:200	1:100	1:50	1:20	1:5
hydrogen peroxide	3% H_2_O_2_	0.0015%	0.006%	0.015%	0.03%	0.06%	0.15%	0.6%
Perisolv (Regedent	0.43% NaClO	0.000215%	0.00086%	0.00215%	0.0043%	0.0086%	0.0215%	0.086%
AG)								
Gum Pareox (Sunstar)	0.12% CHX+ 0.05% CPC	0.00006% CHX+ 0.000025% CPC	0.00024% CHX+ 0.0001% CPC	0.0006% CHX+ 0.00025% CPC	0.0012% CHX+ 0.0005% CPC	0.0024% CHX+ 0.001% CPC	0.006% CHX+ 0.0025% CPC	0.024% CHX+ 0.01% CPC

### 2.2 Cell Culture

MC3T3-E1 cells (CRL-2593, ATCC, Manassas, VA, USA) were cultured in Minimum Essential Medium Alpha (α-MEM, A1049001, Gibco, USA) supplemented with 10% FBS (FCS, 20170–106, Gibco, USA) and 1% penicillin–streptomycin (Gibco, Grand Island, Cat. No 15070–063, NY, USA). HGFs (Passage7, HFIB-G; Provitro, Berlin, Germany) were cultured in DMEM -low glucose (DMEM; Merck, Cat. No D5546, Germany) with 10% FBS, 1% P/S, and 5 mM d-glucose. hBMSCs (Passage 6, Cat. No PT-2501, Lonza, Switzerland) were cultured in DMEM/F12 with Glutamax (Gibco, Grand Island, Cat. No 31331–093, NY, USA) with 10% PIPL, 1% P/S, and 2 IU/ml heparin (LEO Pharma A/Sm Ballerup, Denmark). Cells were incubated at 37 °C in a humidified incubator with 5% CO_2_. Ethical approval was not required for the study, in accordance with local legislation and institutional requirements, because only established, commercially available cells were used.

### 2.3 Cytotoxicity by Lactate Dehydrogenase (LDH) Assay

Cytotoxicity was assessed using the LDH Cytotoxicity Detection Kit (Sigma-Aldrich, Cat. No 11644793001). Cells were seeded in 96-well plates at a density of 7500 cells per well (≈2.3 × 10^4^ cells/cm^2^) and cultured for 24 h. The medium was then replaced with media containing diluted agents and incubated for an additional 2, 12, or 24 h. Supernatants were collected and LDH activity was quantified according to the manufacturer’s instructions. Absorbance was measured at 490 nm using a microplate reader.

### 2.4 Cell Proliferative Viability by CCK8 Assay

Cell proliferative viability was measured using the Cell Counting Kit-8 (CCK-8; Abcam, ab228554). Cells were seeded in 96-well plates at a density of 7500 cells per well (≈2.3 × 10^4^ cells/cm^2^) and cultured for 24 h. The medium was then replaced with diluted agents and incubated for 2, 12, or 24 h. After treatment, the medium was replaced with fresh medium (10% FBS or PIPL) and incubated for an additional 4 h. Aliquots of 100 μL from each well were transferred to a new 96-well plate, and absorbance was measured at 460 nm using a microplate reader, which indirectly reflected cell proliferative activity.

### 2.5 Scanning Electron Microscopy (SEM)

Round coverslips (8 mm in diameter; MENZCB00080RA120, VWR, Germany) were placed in 24-well plates, and cells were seeded at a density of 2.5 × 10^4^ cells per well (≈1.3 × 10^4^ cells/cm^2^). After 24 h, the medium was replaced with diluted agents and incubated for 2, 12, or 24 h. Following treatment, cells were fixed overnight at 4 °C in a solution of 4% paraformaldehyde and 2% glutaraldehyde in HEPES buffer. After that, samples were washed in HEPES buffer, dehydrated through a graded ethanol (30%, 50%, 70%, 90%, twice in 100%, 5 min each round), and immersed in hexamethyldisilazane (HDMS; Sigma-Aldrich, Cat. No. 86944, Burghausen, Germany) for 8 h, allowing slow evaporation. After being sputter-coated with gold, the cell microstructures were examined under an SEM (TM-1000, Hitachi, Tokyo, Japan).

### 2.6 Quantitative Real-Time PCR (qPCR)

TaqMan qPCR probes were used to quantify gene expression. For human cells: β*-actin* (Hs01060665_g1), *BCL2* (Hs00608023_m1), and *BAX* (Hs00180269_m1), and *MCL1* (Hs01050896_m1). For mouse cells: β*-actin* (Mm02619580_g1), *BCL2* (Mm00477631_m1), *BAX* (Mm00432051_m1), and *MCL1* (Mm01257351_g1) (all purchased from ThermoFisher, Cat. No 4331182). Amplification was performed using TaqMan Fast Advanced Master Mix (Thermofisher, Cat. No 4444556). qPCR was conducted on an AriaMx Real-time PCR System (Agilent, USA) in 10 μL reactions containing 3.5 μL PCR-grade water (Sigma-Aldrich, USA, Cat. No W4502), 0.5 μL probe, 5 μL probe mix, and 1 μL template DNA. Cycling conditions were: 50 °C for 2 min (predenaturation), 95 °C for 20 s (polymerase activation), followed by 40 cycles of 95 °C for 1 s (denaturation) and at 60 °C for 20 s (annealing).

### 2.7 Statistical Analysis

Unless otherwise noted, experiments were repeated thrice with at least three to four replicates per group. Data were analyzed using SPSS 28.0 (IBM, USA) and are presented as the mean ± standard deviation. Significant differences between groups were identified using one-way analysis of variance (ANOVA) followed by a Student–Newman–Keuls post hoc test for parametric data or Kruskal–Wallis tests followed by Dunn’s multiple comparison tests for nonparametric data. Differences were considered statistically significant when *p* < 0.05. The data were analyzed and plotted using Prism 10.4.0 (GraphPad Software, USA), with significant differences indicated by different letters (e.g., a, b, c), while groups sharing the same letter are not significantly different (e.g., ab vs b). Figures were formatted using Inkscape (version 1.3.2, Inkscape Project, https://inkscape.org/).

## 3 Results

### 3.1 Cytocompatibility of Debridement Chemicals on MC3T3-E1 Cells

#### 3.1.1 MC3T3-E1 Cells under H_2_O_2_ Treatment

SEM revealed that cells under 0.0015% H_2_O_2_ (1:2000 dilution) maintained normal morphology with multidirectional intercellular connections. In contrast, aggravated shrinkage was observed with H_2_O_2_ concentrations ranging from 0.006% (1:500) to 0.6% (1:5). At 0.006% (1:500) and 0.015% (1:200), cells exhibited irregular protrusions after 2 h of exposure, which diminished and were accompanied by further shrinkage over time. Reduced intercellular connections were observed at concentration of 0.015% (1:200). At 0.06% (1:50) and 0.15% (1:20), the cells became smoother and more spherical. At 24 h, the morphology was no longer maintained in most cells, with evident loss of normal cell shape and integrity (Figure 1A). The SEM morphology of the control group is shown in Figure S1. Proliferative viability was strongly inhibited at concentrations ≥0.006% (1:500), reaching levels comparable to the 1% Triton X-100 positive control (Figure 1B). LDH activity in the supernatant rose in a dose-dependent manner initially, from 0.0015% (1:2000) to 0.15% (1:20). Still, it showed marked reduction at 0.6% (1:5) after 2 h, from 0.15% (1:20) to 0.6% (1:5) after 12 h, and from 0.06% (1:50) to 0.6% (1:5) after 24 h (Figure S2).

**Figure 1:**
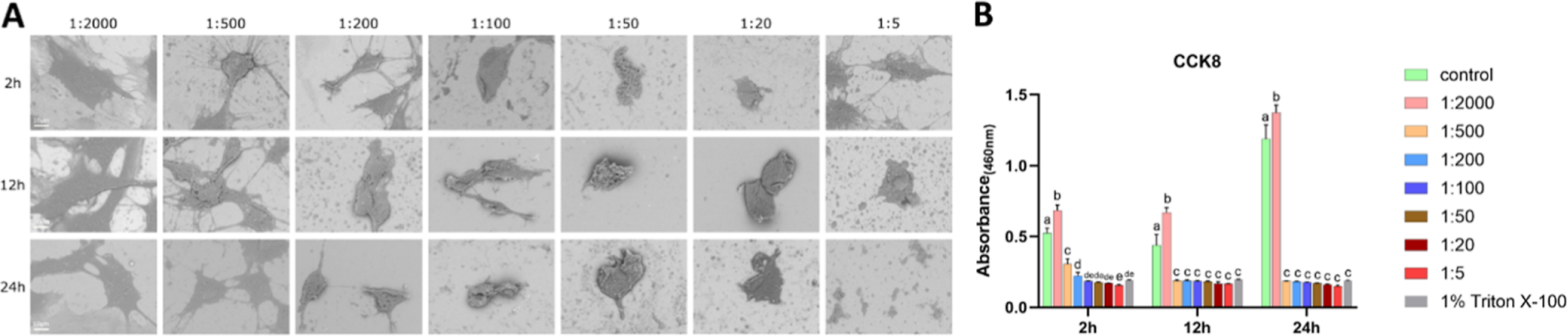
Effects of H_2_O_2_ on MC3T3-E1 cells (A) representative SEM images of MC3T3-E1 cells exposed to different concentrations of H_2_O_2_ for 2, 12, and 24 h; (B) cell proliferative viability under the same conditions. Bars labeled with different letters (e.g., a, b, c) indicate significant differences (*p* < 0.05). Bars sharing the same letter do not differ significantly from each other. Scale bar = 10 μm.

#### 3.1.2 MC3T3-E1 Cells under NaClO Treatment

Under NaClO treatment, SEM showed aggravated shrinkage, increasing from 0.00215% (1:200) to 0.086% (1:5), with the most pronounced changes observed at 0.0215% (1:20) and 0.086% (1:5), where cells adopted a spherical morphology. Shrinkage became more evident with prolonged exposure, being more evident after 24 h than after 2 h, particularly at 0.0215% (1:20) and 0.086% (1:5) (Figure 2A). Unlike H_2_O_2_, which markedly reduced proliferation at concentrations ≥0.006% (1:500), NaClO preserved or even enhanced proliferation at 0.000215% (1:2000) to 0.0086% (1:50). However, compromised viability emerged at higher concentrations, at 0.086% (1:5) after 2 h, and from 0.0215% (1:20) to 0.086% (1:5) after 12/24 h, reaching Triton-comparable levels (Figure 2B). LDH activity in the supernatant declined in a dose-dependent manner, most notably at 0.086% (1:5) (Figure S2).

**Figure 2:**
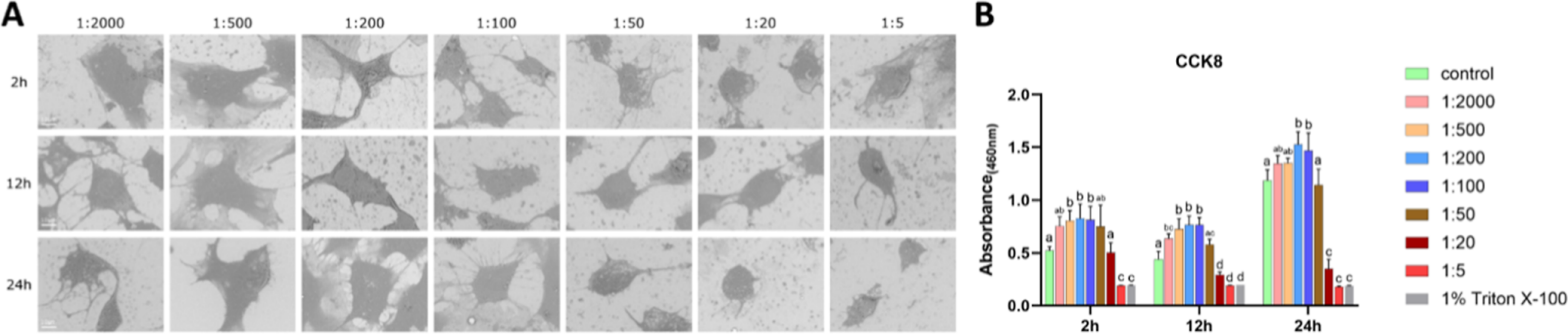
Effects of NaClO on MC3T3-E1 cells (A) representative SEM images of MC3T3-E1 cells exposed to NaClO at gradient dilution ratios for 2, 12, and 24 h; (B) cell viability result. Bars labeled with different letters (e.g., a, b, c) indicate significant differences (*p* < 0.05). Bars sharing the same letter do not differ significantly from each other. Scale bar = 10 μm.

#### 3.1.3 MC3T3-E1 Cells under CHX-CPC Treatment

CHX-CPC induced progressive volume loss and cellular rounding, most evident at 0.006% CHX +0.0025% CPC (1:20). In contrast, at 0.024% CHX +0.01% CPC (1:5), most cells exhibited disrupted membranes and micropore formation across all time points. Even at 0.00006% CHX +0.000025% CPC (1:2000) and 0.00024% CHX +0.0001% (1:500), prolonged exposure intensified shrinkage, with cells volume being smaller at 24 h compared to 2 h (Figure 3A). Proliferative viability was maintained or enhanced at 0.00006% CHX +0.000025% CPC (1:2000) to 0.0024% CHX +0.001% (1:50) at 2 h, 0.00006% CHX +0.000025% CPC (1:2000) to 0.0012% CHX +0.0005% (1:100) at 12 h, and 0.00006% CHX +0.000025% CPC (1:2000) to 0.0006% CHX +0.00025% (1:200) at 24 h, but significantly declined at higher concentrations. Viability was also time-dependent: for example, CHX-CPC at 0.0024% CHX +0.001% (1:50) had no effect at 2 h but reduced viability at 12 h. In comparison, 0.0012% CHX +0.0005% (1:100) was nontoxic at 12 h, but cytotoxic at 24 h (Figure 3B). LDH activity reached Triton-equivalent levels at 0.024% CHX +0.01% CPC (1:5) after 2 h, peaked at 0.0024% CHX +0.001% (1:50) to 0.024% CHX +0.01% CPC (1:5) after 12/24 h (Figure S2).

**Figure 3:**
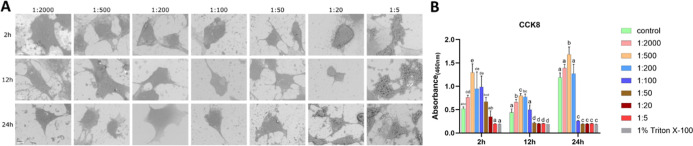
Effects of CHX-CPC on MC3T3-E1 cells (A) representative SEM images of MC3T3-E1 cells exposed to CHX-CPC at gradient dilution ratios for 2, 12, and 24 h; (B) cell viability result. Bars labeled with different letters (e.g., a, b, c) indicate significant differences (*p* < 0.05). Bars sharing the same letter do not differ significantly from each other. Scale bar = 10 μm.

### 3.2 Cytocompatibility of Chemical Compound on HGFs

#### 3.2.1 HGFs under H_2_O_2_ Treatment

Similar to MC3T3-E1 cells, SEM showed progressively increased (dose-dependent) cell shrinkage and reduced intercellular connections at higher concentrations across all time points. For instance, at 2 h, shrinkage was enhanced at higher concentrations, peaking at 0.6% (1:5), at which point cells lost their normal morphology and detached from neighboring cells. With prolonged exposure, even at 0.006% (1:500) and 0.015% (1:200), shrinkage intensified, irregular protrusions disappeared, leaving cells more spherical at 12 and 24h when compared to 2 h (Figure 4A). Similar to MC3T3-E1 cells, proliferative viability of HGFs was strongly inhibited at concentrations ≥0.006% (1:500), comparable to Triton (Figure 4B). LDH activity increased at low concentrations. Still, it declined at 0.6% (1:5) at 2h, from 0.06% (1:50) to 0.6% (1:5) at 12 h, from 0.03% (1:100) to 0.6% (1:5) at 24 h (Figure S2).

**Figure 4:**
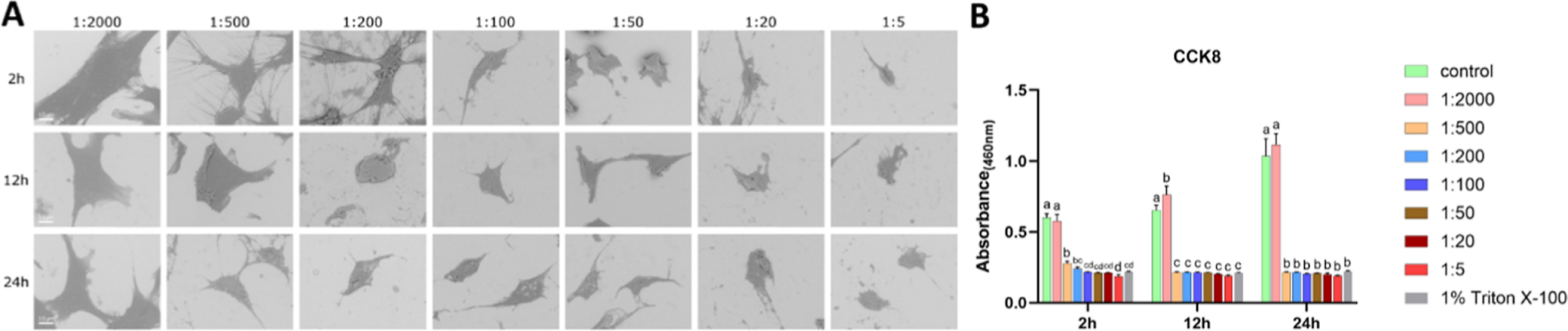
Effects of H_2_O_2_ on HGFs (A) representative SEM images of HGFs exposed to CHX-CPC at gradient dilution ratios for 2, 12, and 24 h; (B) cell viability result. Bars labeled with different letters (e.g., a, b, c) indicate significant differences (*p* < 0.05). Bars sharing the same letter do not differ significantly from each other. Scale bar = 10 μm.

#### 3.2.2 HGFs under NaClO Treatment

NaClO induced dose-dependent shrinkage in HGFs, most severe at 0.086% (1:5), where cells lost intercellular contacts and basic morphology (Figure 5A). Viability was markedly compromised at concentrations ≥0.0086% (1:50). Notably, HGFs were more sensitive to NaClO than MC3T3-E1 cells, as 0.0086% (1:50) NaClO impaired HGF proliferation but had no effect on MC3T3-E1 (Figure 5B). LDH activity decreased significantly from 0.0043% (1:100) to 0.086% (1:5) at 2 h; from 0.0086% (1:50) to 0.086% (1:5) at 12 h; and from 0.0043% (1:100) to 0.086% (1:5) at 24 h (Figure S2).

**Figure 5:**
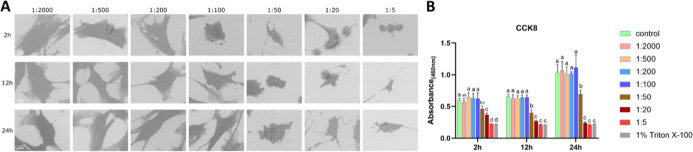
Effects of NaClO on HGFs (A) representative SEM images of HGFs exposed to NaClO at gradient dilution ratios for 2, 12, and 24 h; (B) cell viability result. Bars labeled with different letters (e.g., a, b, c) indicate significant differences (*p* < 0.05). Bars sharing the same letter do not differ significantly from each other. Scale bar = 10 μm.

#### 3.2.3 HGFs under CHX-CPC Treatment

Under CHX-CPC, HGFs morphology changed in both a dose- and time-dependent manner. Shrinkage and reduced intercellular interactions were more pronounced at concentrations ≥0.0012% CHX +0.0005% CPC (1:100), especially at 0.024% CHX +0.01% CPC (1:5). At 0.0012% CHX +0.0005% CPC (1:100), cell volume loss intensified with lmore prolongedexposure, particularly at 24 h (Figure 6A). Similar to MC3T3-E1 cells, CHX-CPC inhibited HGFs proliferative viability at concentrations ≥0.006% CHX +0.0025% CPC (1:20) after 2 h; ≥0.0024% CHX +0.001% CPC (1:50) after 12 h; and ≥0.0006% CHX +0.00025% CPC (1:200) after 24 h (Figure 6B). LDH activity increased significantly from 0.006% CHX +0.0025% CPC (1:20) to 0.024% CHX +0.01% CPC (1:5) at 2 h; from 0.0024% CHX +0.001% CPC (1:50) to 0.024% CHX +0.01% CPC (1:5) at 12 and 24 h (Figure S2).

**Figure 6:**
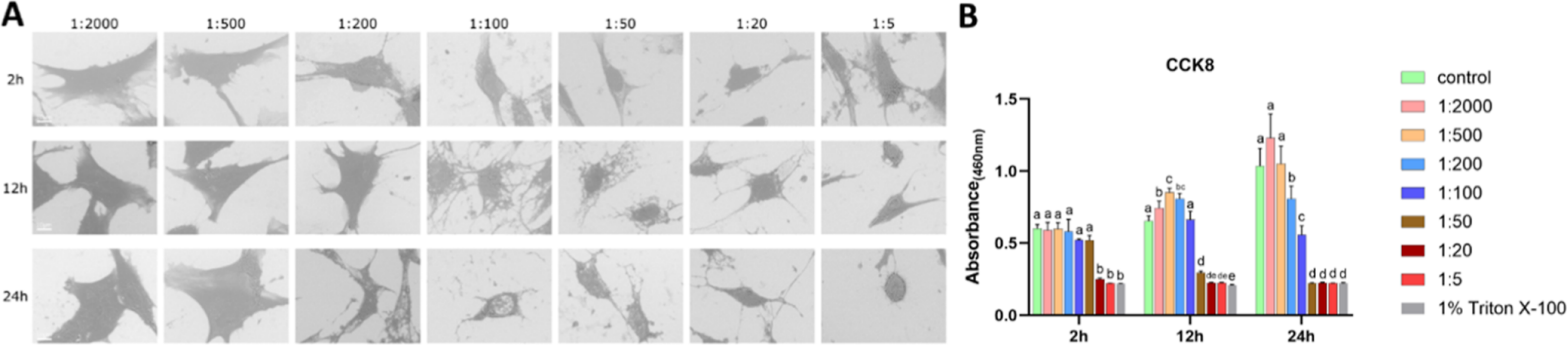
Effects of CHX-CPC on HGFs (A) representative SEM images of HGFs exposed to CHX-CPC at gradient dilution ratios for 2, 12, and 24 h; (B) cell viability result. Bars labeled with different letters (e.g., a, b, c) indicate significant differences (*p* < 0.05). Bars sharing the same letter do not differ significantly from each other. Scale bar = 10 μm.

### 3.3 Cytocompatibility of Chemical Compound on hBMSCs

#### 3.3.1 hBMSCs under H_2_O_2_ Treatment

In hBMSCs, H_2_O_2_ induced dose-dependent shrinkage, most evident at 0.6% (1:5). At 0.06% (1:50), the cell membranes ruffled. At 0.15% (1:20), the nuclei were visible at 2 h. Still, they disappeared with longer exposure (12 and 24 h) (Figure 7A). Unlike MC3T3-E1 and HGFs, hBMSCs maintained viability at control levels from 0.0015% (1:2000) to 0.15% (1:20), with inhibition only observed at 0.6% (1:5), reaching Triton-equivalent levels (Figure 7B). LDH activity in supernatant rose initially. Still, it declined at 0.6% (1:5) (Figure S2).

**Figure 7:**
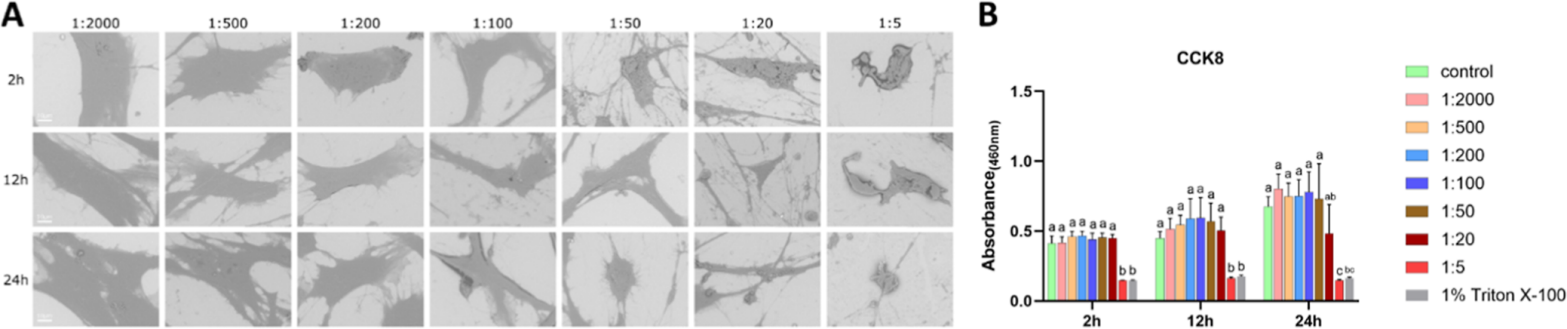
Effects of H_2_O_2_ on hBMSCs (A) representative SEM images of hBMSCs exposed to H_2_O_2_ at gradient dilution ratios for 2, 12, and 24 h; (B) cell viability result. Bars labeled with different letters (e.g., a–c) indicate significant differences (*p* < 0.05). Bars sharing the same letter do not differ significantly from each other. Scale bar = 10 μm.

#### 3.3.2 hBMSCs under NaClO Treatment

NaClO induced cell shrinkage in hBMSCs at 0.0086% (1:50), with worsening at higher concentrations. At 0.0086% (1:50), morphology was partially maintained with some protrusions. Still, at 0.0215% (1:20) and 0.086% (1:5), cell shrank severely and lost intercellular contacts (Figure 8A). Viability inhibition was not only dose-dependent: proliferative viability compromised when concentration ≥0.0215% (1:20) after 2 h, and concentrations ≥0.0086% (1:50) after 12/24 h, but also time-dependent: no inhibition was observed at 0.0086% (1:50) after 2 h, but significant reduction occurred after 12 and 24 h (Figure 8B). Similar to the other two cell types, NaClO maintained control-comparable LDH activity at low concentrations but declined significantly when concentrations ≥0.0086% (1:50) (Figure S2).

**Figure 8:**
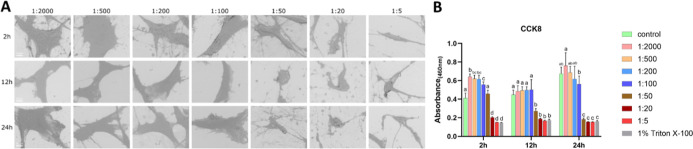
Effects of NaClO on hBMSCs (A) representative SEM images of hBMSCs exposed to NaClO at gradient dilution ratios for 2, 12, and 24 h; (B) cell viability result. Bars labeled with different letters (e.g., a–c) indicate significant differences (*p* < 0.05). Bars sharing the same letter do not differ significantly from each other. Scale bar = 10 μm.

#### 3.3.3 hBMSCs under CHX-CPC Treatment

CHX-CPC induced shrinkage and micropore formation in hBMSCs at 0.006% CHX +0.0025% (1:20) and 0.024% CHX +0.01% CPC (1:5) across all time points. At 0.024% CHX +0.01% CPC (1:5), cell membranes were disrupted, and cells lost their normal morphology. Even at 0.0012% CHX +0.0005% CPC (1:100) and 0.0024% CHX +0.001% CPC (1:50), membrane ruffling and shrinkage became more evident with more prolonged exposure (12 and 24 h) (Figure 9A). Proliferative viability followed the same dose- and time-dependent pattern as the other two cell types: inhibition appeared from 0.006% CHX +0.0025% (1:20) to 0.024% CHX +0.01% CPC (1:5) after 2h; from 0.0024% CHX +0.001% CPC (1:50) to 0.024% CHX +0.01% CPC (1:5) after 12 h; and from 0.0012% CHX +0.0005% CPC (1:100) to 0.024% CHX +0.01% CPC (1:5) after 24 h (Figure 9B). The highest concentrations of CHX-CPC yielded LDH activity comparable to Triton, specifically, 0.024% CHX +0.01% CPC (1:5) at 2 h; from 0.006% CHX +0.0025% (1:20) to 0.024% CHX +0.01% CPC (1:5) at 12 h; and from 0.0024% CHX +0.001% CPC (1:50) to 0.024% CHX +0.01% CPC (1:5) at 24 h (Figure S2).

**Figure 9:**
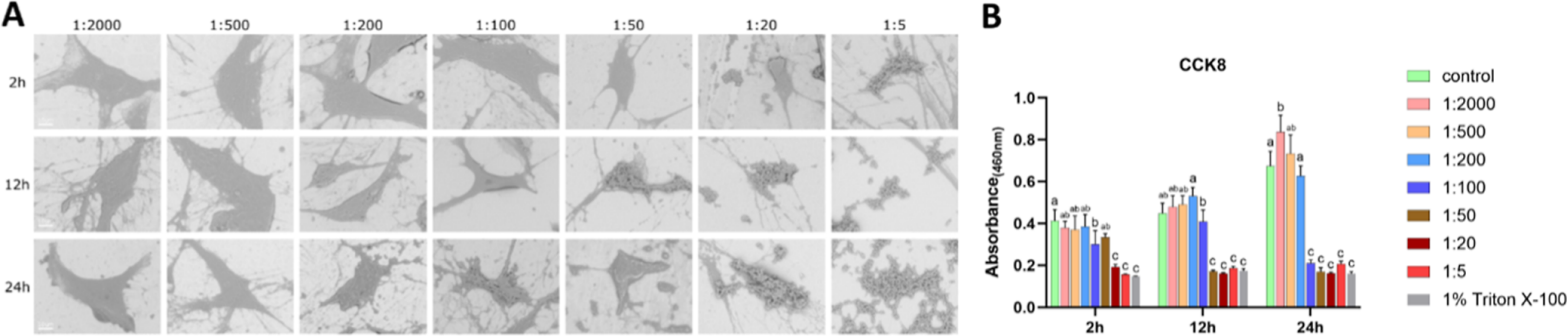
Effects of CHX-CPC on hBMSCs (A) representative SEM images of hBMSCs exposed to CHX-CPC at gradient dilution ratios for 2, 12, and 24 h; (B) cell viability result. Bars labeled with different letters (e.g., a–c) indicate significant differences (*p* < 0.05). Bars sharing the same letter do not differ significantly from each other. Scale bar = 10 μm.

### 3.4 Apoptosis-Related Gene Expression Profiles

#### 3.4.1 Cells under H_2_O_2_ Treatment

In MC3T3-E1 cells, *BCL2* increased at 0.0015% (1:2000) to 0.015% (1:200) but returned to control-level at 0.03% (1:100). *MCL1* was elevated at 0.0015% (1:2000) and decreased under higher concentrations, with *BAX* significantly upregulated when concentrations ≥0.006% (1:500) (Figure 10C). HGFs showed a similar pattern, with elevated *BCL2* and *MCL1* at 0.0015% (1:2000) but reduced at higher concentrations. In contrast, *BAX* increased significantly (Figure 10F). In hBMSCs, *BCL2* was reduced from 0.0015% (1:2000) to 0.006% (1:500), but restored under 0.015% (1:200) and 0.03% (1:100) of H_2_O_2_. *MCL1* was unchanged at 0.0015% (1:2000), increased from 0.006% (1:500) to 0.015% (1:200), and returned to baseline at 0.03% (1:100). *BAX* was significantly upregulated compared with the control (Figure 10I).

**Figure 10:**
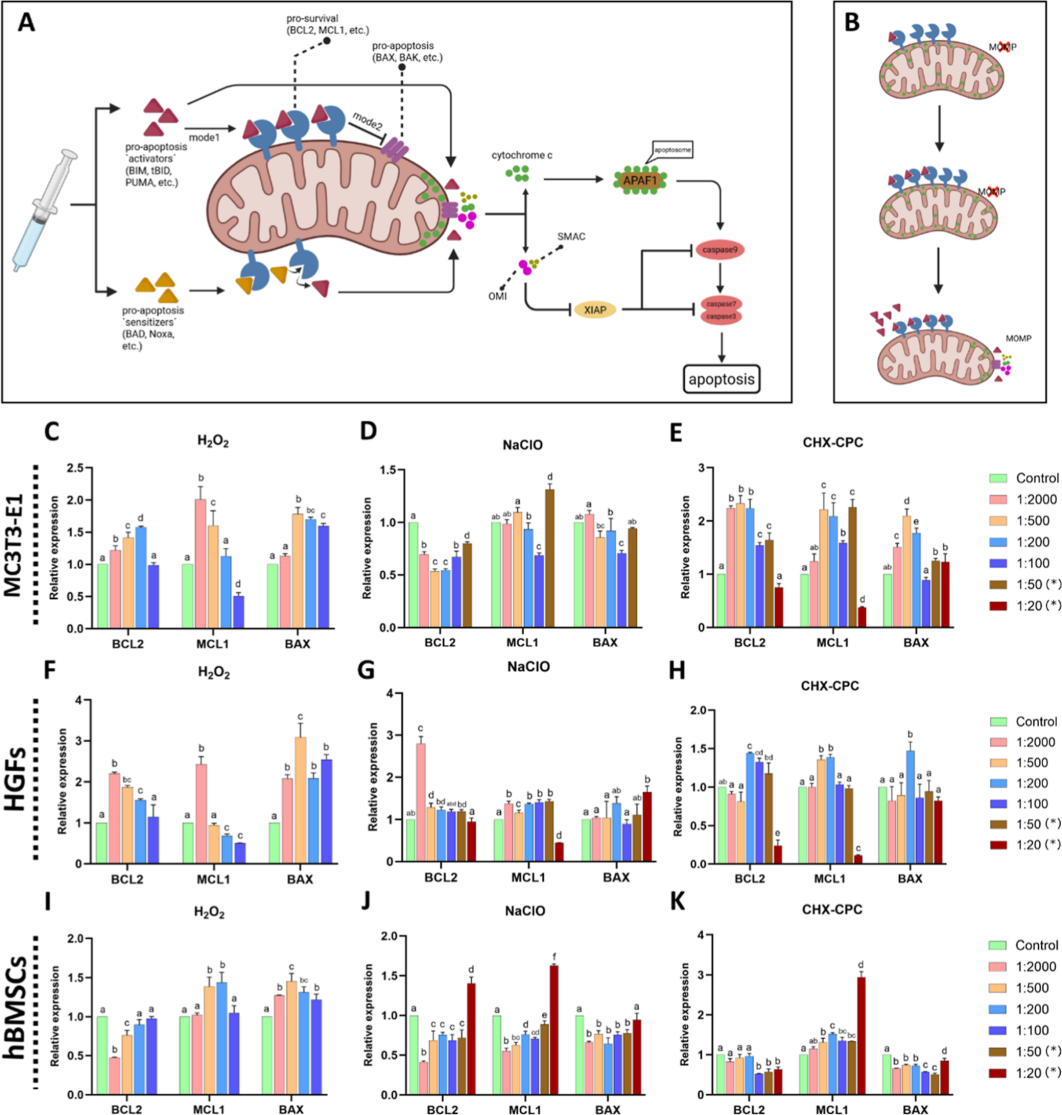
Expression of apoptosis-related genes after chemical compound exposure (A,B) schematic of the intrinsic apoptosis signaling pathway; (C–E) mRNA expression of *BCL2*, *BAX*, and *MCL1* in MC3T3-E1 cells treated with (C)­H_2_O_2_ (D) NaClO, and (E) CHX-CPC; (F–H) Expression of *BCL2*, *BAX*, and *MCL1* in HGFs treated with (F) H_2_O_2_ (G) NaClO, and (H) CHX-CPC; (I–K) Expression of *BCL2*, *BAX*, and *MCL1* in hBMSCs treated with (I) H_2_O_2_ (J) NaClO, and (K) CHX-CPC. The asterisk (*) indicates that too many cells are dead and not enough RNA can be extracted. Bars labeled with different letters (e.g., a–c) indicate significant differences (*p* < 0.05). Bars sharing the same letter do not differ significantly from each other.

#### 3.4.2 Cells under NaClO Treatment

In MC3T3-E1 cells, *BCL2* decreased overall, with a modest rise at high concentrations. *MCL1* decreased at 0.0043% (1:100) but peaked at 0.0086% (1:50), while *BAX* remained comparable to control (Figure 10D). HGFs showed increased *BCL2* at 0.000215% (1:2000). Still, they declined toward control-levels at 0.00215% (1:200). *MCL1* was reduced only at 0.0215% (1:20), with *BAX* significantly upregulated at 0.0215% (1:20) (Figure 10G). In hBMSCs, both *BCL2* and *MCL1* peaked at 0.0215% (1:20), while *BAX* was significantly reduced from 0.000215% (1:2000) to 0.0086% (1:50), and restored at 0.0215% (1:20) (Figure 10J).

#### 3.4.3 Cells under CHX-CPC Treatment

In MC3T3-E1 cells and HGFs, *BCL2* and *MCL1* rose at low concentrations but dropped sharply at 0.006% CHX +0.0025% CPC (1:20) (Figure 10E,H). In hBMSCs, *BCL2* was reduced from 0.0012% CHX +0.0005% CPC (1:100) to 0.006% CHX +0.0025% CPC (1:20), while *MCL1* was strongly upregulated at the highest concentration (dilution of 1:20) (Figure 10K). Under 0.0024% CHX +0.001% CPC (1:50) and 0.006% CHX +0.0025% CPC (1:20), *BAX* was unchanged in MC3T3-E1 cells and HGFs, while suppressed in hBMSCs (Figure 10E–K).

## 4 Discussion

Our previous study, using a titanium coin model, showed that chemical compounds present in commercial debridement agents, even after vigorous saline flushing, impaired cell attachment and proliferation in peri-implant tissue. H_2_O_2_, Perisolv, and Paroex were particularly cytotoxic to hBMSCs, and transcriptional analysis after 2 h of exposure revealed suppression of genes regulating proliferation, adhesion, and antiapoptosis.^10^ A key limitation of this earlier study, however, was the inability to quantify residual concentrations, leaving uncertainty about how dose and exposure time influence biocompatibility. To address this, we systematically exposed MC3T3-E1, HGFs, and hBMSCs to different concentrations of H_2_O_2_, NaClO, and CHX-CPC for 2, 12, and 24 h. The 2 h time point was selected for qPCR to confirm transcriptional changes observed in our earlier work.

All three chemical components reduced viability in a clear dose- and time-dependent fashion. Functionally, hBMSCs were more tolerant only to H_2_O_2_, whereas their viability in response to NaClO and CHX-CPC was similar to that of MC3T3-E1 cells and HGFs.

Although no previous study has directly compared the cytotoxicity of H_2_O_2_ across MC3T3-E1, HGFs, and hBMSCs under identical conditions, threshold values for each cell type have been reported individually. For MC3T3-E1 cells, Dandan et al. demonstrated that 2h exposure to 600 μM H_2_O_2_ (0.002%) markedly reduced cell viability in vitro.^12^ Another study reported that 24 h exposure to 500 μM H_2_O_2_ (0.0017%) similarly impaired MC3T3-E1 viability.^13^ In our study, 0.0015% H_2_O_2_ (441 μM; 1:2000 dilution) did not exert cytotoxic effects on MC3T3-E1 cells, whereas 0.006% H_2_O_2_ (1760 μM; 1:500 dilution) significantly reduced viability, consistent with previously reported cytotoxic thresholds. For HGFs, prior research indicated that even ≤0.0015% H_2_O_2_ (441 μM) for 1 h significantly decreased survival,^14^ suggesting a higher sensitivity than we observed, as 2–24 h exposures at the same concentration (441 μM; 1:2000 dilution) did not reduce HGFs viability in our experiment. For hBMSCs, viability was reported to decline significantly after 8 h at ≥ 200 μM H_2_O_2_ (0.00068%; ∼49% survival),^15^ and even 2 h at 125 μM (0.000425%) reduced viability by ∼42%,^16^ indicating lower toxic thresholds than those observed in our data. These discrepancies, particularly the relatively higher tolerance of HGFs and hBMSCs in our study, are likely attributable to differences in exposure duration, donor source, and passage number.

SEM showed canonical apoptotic morphology that intensified with higher concentrations:

Cell shrinkage, rounding with surface smoothing, membrane ruffling, and fragmentation into small bodies; these features match the classic description of apoptosis.^17,18^ Notably, at 2 h, hBMSCs showed a stronger antiapoptotic transcriptional response, maintaining or upregulating *BCL2* and *MCL1* at high concentrations, despite having a morphology comparable to the other cell types at matched doses, suggesting gene-level buffering that may precede overt structural rescue.

Interestingly, LDH readout declined at the highest concentrations of H_2_O_2_ and NaClO. This does not indicate reduced cytotoxicity; it most likely reflects assay interference, because LDH is unstable under oxidative conditions. Kendig et al. demonstrated that reactive oxidants directly inactivate LDH in culture medium, and that LDH is particularly susceptible to degradation under oxidative conditions, as cellular glutathione is insufficient to protect this enzyme, leading to an artifactual fall in measured activity despite ongoing membrane damage.^19^ Accordingly, the LDH assay is not suitable as a stand-alone cytotoxicity test in the presence of oxidant-based reagents. It can yield false-negative (or inversely dose-dependent) results, especially at high concentrations.

The cytotoxicity of H_2_O_2_ has been extensively reported. Gingival fibroblasts exhibit profound sensitivity, with 100 μmol/L H_2_O_2_ (∼0.03%) reducing proliferation by 80%,^20^ and even lower concentrations (0.00068–0.0010%) impair stemness in rat BMSCs.^21^ In vivo (mouse) rinsing with 0.75–1.5% H_2_O_2_, 4×/day for 2 weeks, occasionally developed erythema and mucosal irritation in healthy volunteers.^22^ This aligns with Abedi et al., who noted that excessive ROS production may hinder tissue repair, as it often causes severe tissue injury and cell damage.^23^ Our results align with these findings, demonstrating that H_2_O_2_ reduces viability and antiapoptotic responses across various cell types in peri-implant tissues. However, it was reported that plaque and salivary bacteria rapidly degrade H_2_O_2_ in vitro, with complete removal of up to ∼300 mmol/L (∼1%) within 15 min^24^ This may explain why low-concentration H_2_O_2_ in dentifrices (0.75%) is generally safe for oral hard and soft tissues, with reported benefits in plaque control and wound healing.^25^ Hence, while our in vitro data confirm cytotoxic potential, the clinical relevance requires validation in clinical settings. Variations in bacterial species and numbers around dental implants may significantly affect the potential for H_2_O_2_ consumption, making it difficult to determine the exact “safe dose” for H_2_O_2_ application. It may differ significantly for patients with varying oral hygiene and genetic backgrounds.^26,27^

NaClO, a potent oxidizing and proteolytic agent, is widely used for root canal irrigation due to its capacity to dissolve necrotic tissue and disrupt biofilms.^28^ Its cytotoxicity arises from reactive species such as OCl^–^, HOCl, and OH-, which elevate pH, while HClO also directly interacts with DNA bases, resulting in oxidative damage and, eventually, cell death.^29–31^ Although complications may occur with the inadvertent extrusion of concentrated NaClO,^32^ its clinical use persists due to superior antimicrobial activity.^33^ In vitro, NaClO reduces hBMSC viability in a concentration- and time-dependent manner, with significant inhibition at 0.05% after 2 h,^34^ which is similar to our findings that proliferative viability was significantly inhibited even at 0.02% for 2 h (1:20 dilution of NaClO). These results highlight the need for cautious clinical application and further in vivo evaluation.

CHX, a cationic diphenyl compound with broad-spectrum bactericidal activity, is widely used in periodontal and peri-implant therapy.^35^ Its cytotoxicity, however, is well documented. Gisele et al. showed that CHX induces endoplasmic reticulum stress, leading to apoptosis or necrosis. Low concentrations (0.000125% and 0.001%) slightly increased *BCL2* in murine fibroblasts, whereas ≥0.004% caused necrotic death due to membrane disruption.^36^ M. Giannell et al. further demonstrated dose- and time-dependent cytotoxicity in gingival fibroblasts and alveolar osteoblasts, mediated by mitochondrial dysfunction, intracellular Ca^2+^ overload, and oxidative stress, cautioning against its direct use in regenerative procedures.^37^ Goldschmidt et al. reported that even brief exposure (10 min) to 0.004% CHX impaired protein synthesis,^38^ while James et al. confirmed significant toxicity on human fibroblasts, myoblasts, and osteoblasts at concentrations up to 100-fold below the clinical use level (2%).^5^ And Marzena et al. showed that 0.002% CHX for 15 min did not impair HGFs proliferation or morphology, but ≥0.04% markedly reduced viability.^39^ Although the exposure time and dilution medium differ (CHX is diluted in FBS-free solutions), consistent with these findings, our results confirmed dose-dependent cytotoxicity of CHX-CPC, underscoring the potential risk of impaired osseointegration and soft tissue healing if residual CHX persists.

CPC is a quaternary ammonium compound with amphiphilic properties and broad-spectrum antimicrobial activity, widely used in dentistry, particularly in mouthwashes alone or in combination with CHX, and generally associated with fewer side effects than CHX.^40,41^ In vitro studies simulating clinical use have reported limited cytotoxicity. For instance, Geneviève et al. showed that 1 min exposure to 0.05% CPC with 0.2% NaF (1/4 dilution) did not significantly affect epithelial cell viability,^42^ and Heitor et al. found no significant toxicity in L929 murine fibroblasts after 48 h exposure to 0.0195% CPC.^43^ By contrast, Doris et al. reported cytotoxicity in retinal pigment epithelial cells and keratinocytes at micromolar concentrations (150-fold lower than 0.05%), although this involved 48 h exposure, well beyond clinically relevant conditions.^44^ Similarly, Mustafa et. al showed that 2 min exposure to various dilutions of commercial mouthwashes (Colgate Plax, Oral B Proexpert) reduced fibroblast viability;^45^ however, these products contain additional components (e.g., sorbitol, poloxamer 407, propylene glycol, sodium fluoride, sodium saccharin, etc.), and the use of murine fibroblasts limits direct clinical interpretation. Consequently, we assume that the cytotoxicity of CHX-CPC is primarily derived from CHX in the present study.

Our previous work demonstrated that oxidant-based agents (H_2_O_2_, NaClO) activate the intrinsic apoptotic pathway,^10^ a process regulated by the BCL-2 family of proteins. In this system, pro-survival proteins such as BCL-2 and MCL1 oppose pro-apoptotic proteins such as *BAX* and *BAK*. Apoptosis occurs when the balance shifts, allowing *BAX* and *BAK* to oligomerize and form macro-pores in the mitochondrial outer membrane, thereby permeabilizing the mitochondrial outer membrane (MOMP) and triggering caspase activation. Since MOMP is the critical step at which a cell irreversibly commits to undergoing apoptotic cell death, it represents a cellular “point of no return”.^46^ Thus, a shift toward lower *BCL2*/*MCL1* with higher *BAX*/*BAK* reflects a pro-apoptotic BCL-2 family balance, favoring *BAX*/*BAK* activation, MOMP, and commitment to caspase-dependent apoptosis. Our results suggest that at low-concentration agents, cells upregulate pro-survival proteins in response to apoptotic signals, but at high concentrations, this defense is overwhelmed, enabling MOMP and subsequent apoptosis (Figure 10B).^47^ Interestingly, BH3 (BCL2-homology-3)-only proteins show selective preferences: NOXA primarily targets *MCL-1*, while *BAD* favors *BCL-2* and *BCL-XL*.^48^ Structural studies have shown that distinct amino acid substitutions underlie the binding specificity of *MCL1* and *BCL-XL*.^49^ This may explain our finding that, under the highest CHX-CPC exposure, *MCL1* was strongly upregulated, while *BCL2* declined, with *BAX* remaining suppressed, likely due to MCL1’s buffering effect. These data highlight distinct regulatory roles for *MCL1* and *BCL2* despite their shared antiapoptotic function. Cell-type-specific differences were also evident.^48^ hBMSCs showed greater resistance to cleaning agents than MC3T3-E1 and HGFs, maintaining or even increasing *BCL2* and *MCL1* expression under concentrated NaClO, and strongly upregulating *MCL1* under CHX-CPC. One possible explanation is hBMSĆs lower proliferation rate, which is associated with reduced apoptotic priming, while rapidly dividing cells are generally more prone to apoptosis, such as MC3T3-E1 and HGFs.^50^

Several limitations of this study should be acknowledged. First, peri-implant tissues are composed of diverse cell populations beyond those examined here. Future studies should therefore include peri-implant epithelial cells, which form semidesmosome attachments and an internal basal lamina, essential for epithelial sealing in the transmucosal area, as well as immune cells such as polymorphonuclear leukocytes and macrophages, which play key roles in bacterial eradication and angiogenesis.^2^ Second, our in vitro model cannot replicate the vascularized and inflammatory environment of peri-implant tissues, where local and systemic immune responses shape cytocompatibility.^5^ Moreover, clinical peri-implantitis is characterized by complex biofilms dominated by anaerobic pathogens (*Porphyromonas gingivalis*, *Tannerella forsythia*, *Treponema denticola*, and *Fusobacterium nucleatum*), whose coexistence in an anaerobic, inflammatory niche may profoundly alter peri-implant cell responses to chemical residuals, for instance, oral bacteria in vivo rapidly degrade H_2_O_2_, likely diminishing its biological impact compared with in vitro conditions.^51^ Third, technical factors must be considered: fetal bovine serum in the culture medium can attenuate the cytotoxicity of H_2_O_2_, NaClO, and CHX.^52–54^ In this study, MC3T3-E1 cells and HGFs were cultured with FBS, whereas hBMSCs were maintained with platelet lysate, whose protective effects remain uncertain, thus, cross-cell comparisons, particularly regarding relative apoptosis resistance-should be made cautiously. Highest concentration dilution (1:5) in medium reduced the FBS from 10% to ∼8%, which may weaken protective support and potentially exaggerate cytotoxicity. Besides, primary human cells can exhibit donor-to-donor variability, confirmation of these findings using primary cells from multiple donors will be important before broader generalization.^55,56^ These limitations underscore the need for more physiologically relevant models to evaluate the biological effects of residual chemical cleaning agents under clinically realistic conditions.

In summary, this study demonstrates that chemical debridement agents exert cytotoxic effects, impairing the proliferative viability of cells in peri-implant tissues in a time- and dose-dependent manner. In light of these findings, future therapeutic strategies for peri-implant mucositis and peri-implantitis should not rely solely on thorough chemical debridement but also incorporate biomaterials that activate tissue healing and regeneration.

## 5 Conclusion

This study demonstrated that residual chemical debridement agents in peri-implant pockets exert time- and dose-dependent cytotoxic effects on peri-implant cells under the conditions tested. However, all cell populations were negatively affected by agents that were not sufficiently removed. Within the limitations of this in vitro model, these findings underscore the importance of thoroughly removing residual decontamination agents to maintain peri-implant cell viability, implant biocompatibility, and a healing environment, as well as the need for additional biomaterials to stimulate healing and regeneration. From a translational perspective, our results support the rationale for rigorous rinsing after implant surface decontamination to facilitate peri-implant healing following peri-implantitis treatments. At the same time, further validation in more complex in vivo models is required.

## Supplementary Material



## Notes

Figure S1. Morphology of cells in the control group. SEM images of MC3T3-E1, HGFs, and hBMSCs cultured under control conditions (medium only) for 2, 12, and 24 h. Scale bar = 10 μm. Figure S2. LDH activity in the culture supernatant. LDH activity measured in the culture supernatant of MC3T3-E1, HGFs, and hBMSCs following exposure to graded dilutions of H2O2, NaClO, and CHX-CPC for 2, 12, and 24 h (PDF)
