# Supplementary material for: Residuals of Chemical Cleaning Agents Impair Peri-Implant Cell Viability: An in Vitro Study
Source: ACS Biomater Sci Eng. 2026 Jan 13;12(2):878–88. doi: 10.1021/acsbiomaterials.5c01777 (PMC12892246; doi:10.1021/acsbiomaterials.5c01777)
Supplement: Supplementary file 1 [file ab5c01777_si_001.pdf]

Supporting information for publication

Residuals of chemical cleaning agents impair peri-implant cell viability: an in vitro study

Authors

Qiang Wang [a], Håvard Jostein Haugen [a*], Dirk Linke [b], Ståle Petter Lyngstadaas [a], Qianli Ma [a*]

Affiliations

[a] Department of Biomaterials, Faculty of Dentistry, University of Oslo, 0455 Oslo, Norway

[b] Department of Biosciences, Faculty of Natural Sciences, University of Oslo, 0316 Oslo, Norway

Corresponding author

*Håvard Jostein Haugen and *Qianli Ma are listed as co-corresponding authors.

*Email: h.j.haugen@odont.uio.no;Phone: +47 91641610

*Email: qianlima@odont.uio.no; Phone: +47 46274377

Page S1: Cover page

Page S2: Figure S1

Page S3: Figure S2

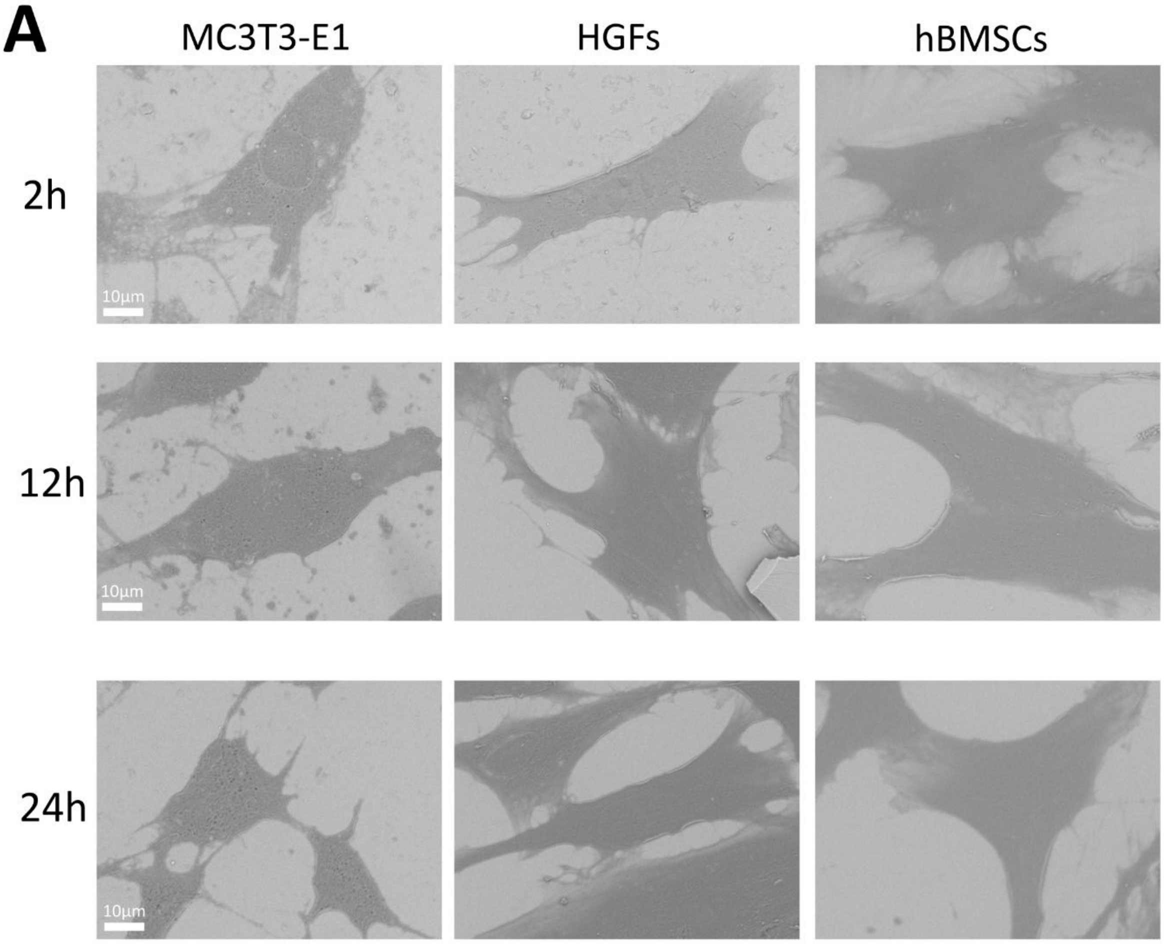

Figure S1. Morphology of cells in the control group. SEM images of MC3T3-E1, HGFs, and hBMSCs cultured under control conditions (medium only) for 2, 12, and 24 hours. Scale bar =10 μm.

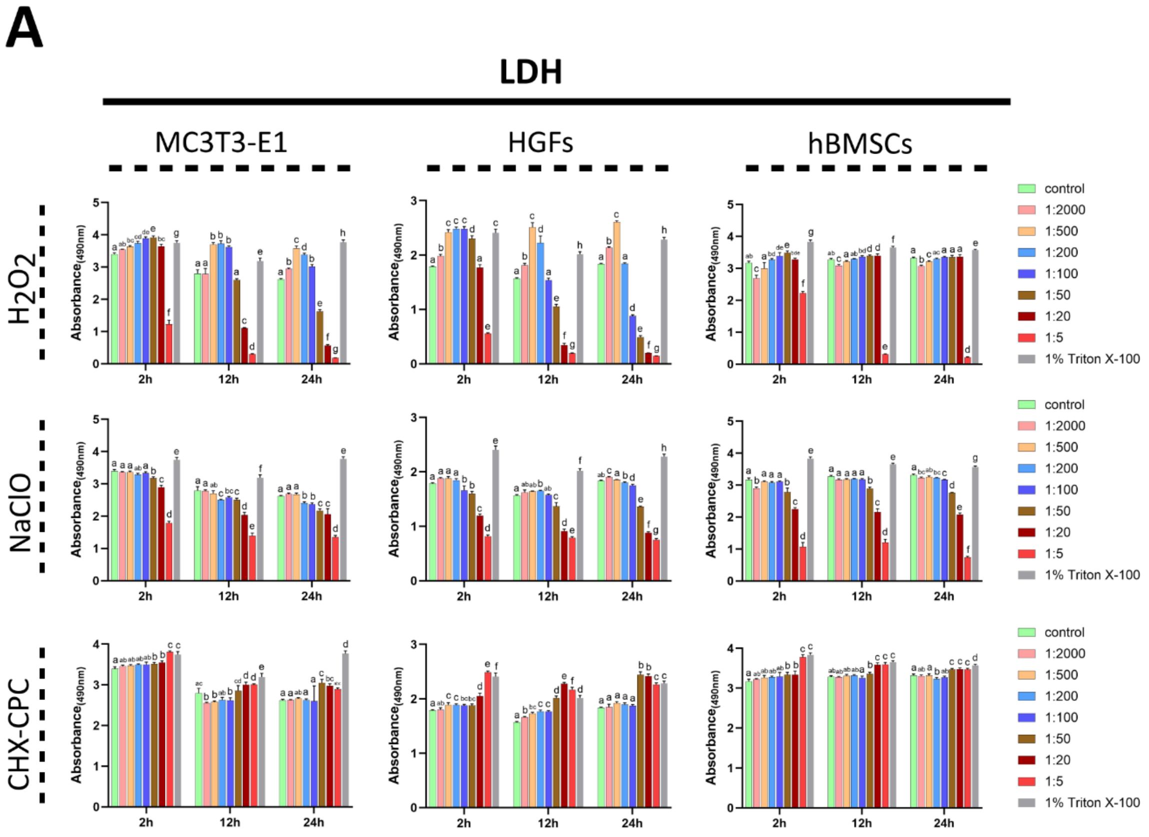

Figure S2. LDH activity in the culture supernatant. LDH activity measured in the culture supernatant of MC3T3-E1, HGFs, and hBMSCs following exposure to graded dilutions of H_2O_2, NaClO, and CHX-CPC for 2, 12, and 24 hours.
